# Compensatory remodeling of a septo-hippocampal GABAergic network in the triple transgenic Alzheimer’s mouse model

**DOI:** 10.1186/s12967-023-04078-7

**Published:** 2023-04-15

**Authors:** Connor M Wander, Ya-Dong Li, Hechen Bao, Brent Asrican, Yan-Jia Luo, Heather A Sullivan, Tzu-Hao Harry Chao, Wei-Ting Zhang, Samantha L Chéry, Dalton S Tart, Ze-Ka Chen, Yen-Yu Ian Shih, Ian R Wickersham, Todd J Cohen, Juan Song

**Affiliations:** 1 Department of Pharmacology, University of North Carolina, Chapel Hill, NC 27599 USA; 2 Neuroscience Center, University of North Carolina, Chapel Hill, NC 27599 USA; 3 Songjiang Research Institute, Songjiang hospital, Shanghai Jiao Tong University School of Medicine, Shanghai, 201699 China; 4 Department of Anaesthesiology, Shanghai Ninth People’s Hospital, Shanghai Jiao Tong University School of Medicine, Shanghai, 201699 China; 5 McGovern Institute for Brain Research, Massachusetts Institute of Technology, Cambridge, MA 02139 USA; 6 Department of Neurology, University of North Carolina, Chapel Hill, NC 27599 USA; 7 Biomedical Research Imaging Center, University of North Carolina, Chapel Hill, NC 27599 USA

**Keywords:** Alzheimer’s disease, Septo-hippocampal GABAergic network, Dentate gyrus, Medial septum

## Abstract

**Background:**

Alzheimer’s disease (AD) is characterized by a progressive loss of memory that cannot be efficiently managed by currently available AD therapeutics. So far, most treatments for AD that have the potential to improve memory target neural circuits to protect their integrity. However, the vulnerable neural circuits and their dynamic remodeling during AD progression remain largely undefined.

**Methods:**

Circuit-based approaches, including anterograde and retrograde tracing, slice electrophysiology, and fiber photometry, were used to investigate the dynamic structural and functional remodeling of a GABAergic circuit projected from the medial septum (MS) to the dentate gyrus (DG) in 3xTg-AD mice during AD progression.

**Results:**

We identified a long-distance GABAergic circuit that couples highly connected MS and DG GABAergic neurons during spatial memory encoding. Furthermore, we found hyperactivity of DG interneurons during early AD, which persisted into late AD stages. Interestingly, MS GABAergic projections developed a series of adaptive strategies to combat DG interneuron hyperactivity. During early-stage AD, MS-DG GABAergic projections exhibit increased inhibitory synaptic strength onto DG interneurons to inhibit their activities. During late-stage AD, MS-DG GABAergic projections form higher anatomical connectivity with DG interneurons and exhibit aberrant outgrowth to increase the inhibition onto DG interneurons.

**Conclusion:**

We report the structural and functional remodeling of the MS-DG GABAergic circuit during disease progression in 3xTg-AD mice. Dynamic MS-DG GABAergic circuit remodeling represents a compensatory mechanism to combat DG interneuron hyperactivity induced by reduced GABA transmission.

**Supplementary Information:**

The online version contains supplementary material available at 10.1186/s12967-023-04078-7.

## Background

Age-related dementia affects almost 10% of people in the United States [1]. Despite enormous healthcare advances in recent times, the number of individuals living with dementia continues to grow significantly, leading to an enormous healthcare burden on patients and their families. Alzheimer’s disease (AD), the most prevalent type of dementia, is characterized by a progressive loss of memory that cannot be efficiently managed by currently available AD therapeutics. Most of the treatments for AD that have the potential to improve memory functions work by leveraging neural networks to protect circuit integrity [2–8].

Currently, there remains a limited understanding of the vulnerable circuits and their dynamic remodeling during AD progression [9]. Most research has focused on understanding the relationship between memory impairments and the formation of pathological hallmarks (Aβ plaques and tau neurofibrillary tangles) seen in the late stages of AD [10, 11]. In contrast, the early stages of AD have received relatively less attention, despite the identification of synaptic and circuit phenotypes as major correlates of cognitive impairments in both human patients and mouse models [12–14]. How neural circuits remodel during AD progression remains largely undefined. Therefore, identifying vulnerable circuits is critically important for understanding the development and progression of AD to facilitate the development of properly timed therapeutic strategies.

Spatial memory decline has been known to be an early clinical sign of AD [ 15, 16], for which the dentate gyrus (DG) has a crucial role [ 11, 17]. Accumulating evidence has revealed that dysfunction of hippocampal interneurons is associated with memory deficits in mouse models of AD [ 7, 18–22], highlighting the importance of the hippocampal inhibitory network in regulating cognitive functions in AD. Despite these revelations, the mechanism of DG interneuron dysfunction and the potential remodeling of these interneurons in response to progressive AD pathology have not been extensively examined [ 23, 24]. Interestingly, using rabies-based monosynaptic retrograde tracing, we found DG interneurons exhibit high connectivity with medial septum (MS) GABAergic neurons. Moreover, in vivo fiber photometry recording revealed correlated activities of DG and MS GABAergic neurons during spatial memory encoding. These findings prompted us to examine the MS-DG GABAergic circuit in the context of AD progression.

Enriched MS GABAergic projections to the hippocampus [25] influence theta rhythm, adult hippocampal neurogenesis [26, 27], object exploration [28], and spatial memory [ 29, 30]. Our group previously demonstrated that ablation of MS GABAergic neurons induces DG hyperexcitability [26], suggesting a critical role of MS GABAergic neurons in controlling the inhibitory tone of the DG network. Additionally, degeneration of MS GABAergic neurons in late-stage AD has been reported in both AD mouse models [31–33] and human patients [34]. Ample evidence has shown that neural circuits undergo extensive remodeling well before actual neurodegeneration occurs [9, 35]. Therefore, identifying AD-vulnerable neural circuits that undergo remodeling during early AD may provide critical information on the treatment window by targeting these neural circuits before AD pathology worsens and neuronal death accumulates.

To address whether the MS-DG GABAergic circuit undergoes remodeling during AD progression, we employed the well-established triple transgenic AD model, 3xTg-AD [36, 37]. The 3xTg-AD mice harbor the AD pathological hallmarks of amyloidosis and tauopathy, and exhibit relatively slow progression of AD [38], thus allowing the study of circuit remodeling during well-defined early and late AD stages. In this study, we first performed rabies-based monosynaptic retrograde tracing and confirmed high anatomical connectivity between MS and DG GABAergic neurons. Then we performed fiber photometry recordings and showed that MS and DG GABAergic neurons are correlated during spatial memory encoding. Furthermore, using a combination of circuit tracing, slice electrophysiology, and fiber photometry, we reported dynamic structural and functional remodeling of the MS-DG GABAergic circuit during AD progression.

## Methods

### Mouse model

Vgat-ires-cre knock-in (Vgat-Cre) mice were obtained originally from The Jackson Laboratory (RRID: IMSR_JAX:028862), and back-crossed with C57BL/6J mice for 10 + generations. Triple-transgenic mice (3xTg-AD; Jackson Laboratory (RRID: MMRRC_034830-MU))harbor expression of human transgenes with familial mutations associated with AD, including PSEN1 M146V, APP Swedish, and MAPT P301L. Homozygous Vgat-Cre animals were crossed with 3xTg-AD animals over successive generations to generate Vgat-Cre+/-::3xTg-AD+/+ animals (Vgat-AD) or Vgat-Cre+/- littermate controls (Vgat-WT) on a mixed genetic background [39].

Animals were housed in sterilized cages in groups of 2–5 same-sex littermates per cage. Animals were kept under 12 h light cycles with ZT0 = 7 pm and given food and water ad-libitum. All behavioral and surgical procedures were performed at consistent times when possible. Both male and female animals were used in this study at equal proportions, however, females were excluded from the study if used as breeders, and males were excluded from the study if infighting or scarring was observed. All animal procedures were performed in accordance with the Institutional Animal Care and Use Committee’s (IACUC) regulations at University of North Carolina, Chapel Hill.

### Stereotaxic surgeries

For retrograde and anterograde tracing experiments, Vgat-WT and Vgat-AD mice were anesthetized using 1.5% isoflurane and oxygen at 0.8 L/min. AAVs were injected via a Hamilton microsyringe (Cat no. 33GA) and a Harvard Apparatus infusion pump at 50 nl/min using coordinates described below. The syringe and needle apparatus were left for 10 min after infusion to permit full viral diffusion before removal and surgical wound closing with Vetbond Tissue Adhesive (1469c). All coordinates were selected using “The Mouse Brain in Stereotaxic Coordinates”. Animals were observed for at least 30 min post-recovery with heat support to maintain body temperature before being returned to the home cage.

### Tissue collection and immunofluorescence

Animals were anesthetized with 5% isoflurane in oxygen and transcardially perfused with cold 1X PBS and 4% PFA for endpoint analyses. Brain tissue was removed and fixed for 24 h in 4% PFA at 4 ℃, cryoprotected in 30% sucrose for > 48 h at 4 ℃, and sectioned at 40 µM on a Leica microtome before storage at −20 ℃ in ethylene glycol antifreeze. Immunofluorescence staining was conducted on free floating sections as follows: sections were rinsed 3x with 1X TBS, permeabilized in 2% Triton-X100 in 1X TBS for 30 min, rinsed 4x with 1X TBS and then blocked in 2% donkey serum in 1X TBS for 50 min. Primary and secondary antibodies were diluted in blocking solution and incubated with tissue for 48 h and 16 h, respectively, with the following dilutions: APP (6E10) 1:500, GABA 1:1000, GFP 1:250, GFAP 1:1000, RFP 1:250. All secondaries were used at 1:500 dilution.

### Retrograde tracing

For RV based retrograde tracing, mice were injected with a 50/50 mixture of pre-diluted AAV1-syn-FLEX-splitTVA-EGFP-tTA (7.6E10 gc/ml) and AAV1-TREtight-mTagBFP2-B19G (6.5E11 gc/ml) [40–42] into the left DG at AP: −2.0 mm, ML: +1.4 mm, DV: −2.0 mm. After 2 weeks, animals received a second injection of 250 nl pseudo-typed rabies virus RV∆G-4mCherry (EnvA)[43] (using the same coordinates. Animals were then transferred to a quarantined cubicle for special housing and monitoring. Seven days post rabies injection, animals were perfused and brain tissues were collected at a final time point of 20 and 56 weeks of age. Sectioning was performed as described above.

The area of mCherry was measured based on a recent publication [44].Whole-brain free-floating frozen coronal sections were retrieved from antifreeze and plated on superfrost plus charged slides. Every sixth section was retrieved and plated to encompass the entire brain from the olfactory bulb to the cerebellum. Slides were air dried for 45 min, DAPI stained, cover slipped, and allowed to dry overnight. Full slides were scanned on the Aperio Scanscope FL at the Translational Pathology Labs (TPL) imaging core (UNC). Digital image files were then uploaded to TPL servers and analyzed via TPL’s eSlide manager, where investigators were blinded to genotypes. Regions of interest were defined according to standard anatomical maps. Images were first thresholded and mCherry area was quantified within the regions of interest (ROIs) across the whole brain. Finally, the connectivity ratio of inputs to the DG interneurons was calculated as the area of mCherry within each ROI (input brain region), normalized to the area of mCherry starter cells in the DG.

### Anterograde tracing

Anterograde tracing experiments were conducted by injecting Vgat-WT and Vgat-AD mice with AAV5-ef1a-DIO-YFP into the medial septum [AP: +0.75 mm, ML: 0.00 mm, DV: −3.75 mm]. The VGAT promoter-driven Cre recombinase inverts sequences at loxP sites within DIO-YFP to express YFP exclusively in MS GABA neurons. Mice were anesthetized and injected as described above, and allowed 2 weeks of recovery in groups of littermates before perfusion and tissue collection. Injections were timed such that mice were perfused at 6, 10, and 14 months of age. MS tissue sections were manually counted to determine YFP^+^ cell density to confirm target selectivity and viral expression, which was used as an exclusion criterion prior to the analysis of DG projections.

YFP signal in tissue sections was captured via confocal microscopy (FV3000RS at 40x) and then reconstructed using a 3D surface-surface contact algorithm in IMARIS (Oxford). IMARIS analysis volume was created via a manually drawn ROI of the hilus using the DAPI channel, which was then extruded to the full section volume (40 μm). Thresholding was optimized to retain fine process structure while removing the background and then standardized for all following batch protocols between genotypes. 3D reconstruction yielded > 2000 objects per image on average, each with individual volumes which were summed in Microsoft Excel and then normalized by total hilar ROI volume. Resulting values for each serial section were then averaged per animal and then normalized by MS-YFP + cell density. All projection and immunofluorescence analyses were conducted each 6th serial section for MS and DG. YFP + cells in the MS were manually counted across 40 confocal single planes in ImageJ. Projection density was normalized by hilar ROI volume and YFP^+^ starter cell density (cells/MS ROI area x 40 μm). All image analysis was performed blind to experimental genotype.

### Slice electrophysiology

In vitro electrophysiology experiments were performed as follows: 4–6 weeks after recovery from AAV injections into the MS, mice were anesthetized with 5% isoflurane in oxygen and transcardially perfused with ice-cold NMDG-based aCSF (N-methyl-D-glucamine) saturated with 95% O2 and 5% CO2 and containing the following supplements (in mM): 25 glucose, 20 HEPES, 10 MgSO4, 5 sodium ascorbate, 3 sodium pyruvate, 2.5 KCl, 2 thiourea, 92 NMDG, 30 NaHCO3, 1.25 NaH2PO4, and 0.5 CaCl2 (pH 7.3, 305–315 mOsm). Brains were rapidly removed before acute coronal slicing of hippocampal sections at 280 μm using a vibratome (VT1200, Leica, Germany). Sections were then equilibrated at 34.5 °C for 8 min and maintained in a holding chamber containing HEPES aCSF (in mM): 92 NaCl, 30 NaHCO3, 25 glucose, 20 HEPES, 5 sodium ascorbate, 3 sodium pyruvate, 2.5 KCl, 2 thiourea, 2 MgSO4, 2 CaCl2, and 1.25 NaH2PO4 (pH 7.3, 305–315 mOsm) at room temperature for at least 1 h before recording.

For all experiments, slices were placed in a submerged slice chamber and continuously perfused with ACSF at 2 ml/min maintained at 32 °C with an in-line heater system (TC-324B; Warner Instruments). Slices were visualized on a fixed-stage upright microscope (Olympus BX51WI) equipped with ×10 and ×60 objectives using differential interference contrast (DIC) optics and infrared illumination. Epifluorescence illumination was used to identify ChR2-EYFP projection fibers from MS GABAergic neurons.

To activate ChR2 in acute slices, 473 nm LED light was focused onto the back aperture of the 60× water immersion objective to produce collimated whole-field illumination. Square pulses of LED light (20 Hz or 10 Hz at 5 ms, for 20 s) were delivered every 60 s.

Electrophysiological recordings were conducted in a whole-cell configuration with a multi-clamp 700B amplifier (Axon Instruments). Signals were filtered at 1 kHz and sampled at 10 kHz via the Digidata 1440 A (Axon Instruments), data was acquired using pClamp 10.3 (Axon Instruments). Access resistance of recordings was monitored throughout the experiment. Data were discarded if the access resistance changed by > 20%. For intrinsic membrane property recording, granule cells located in the middle to outer layer of DG were recorded in current-clamp mode. A K-gluconate-based intracellular solution was used (in mM): 130 potassium gluconate, 2 NaCl, 4 MgCl2, 20 HEPES, 0.5 EGTA, 4 Na-ATP and 0.4 Na-GTP, pH 7.3 and ~ 300 mOsm. To test neuronal input resistance during recordings in current clamp mode, hyperpolarizing current pulses (20 pA, 200 ms) were applied to the neurons.

To record spontaneous inhibitory postsynaptic currents (sIPSCs), a cesium chloride-based intracellular solution was used (in mM): 140 CsCl, 10 HEPES, 0.3 EGTA, 5 QX-314-Br, 4 Mg-ATP, 0.3 Na-GTP, and 10 Phosphocreatine disodium salt hydrate. sIPSCs were recorded in voltage clamp (holding at -65 mV) and isolated by blocking ionotropic glutamatergic transmission with 6,7-dinitroquinoxaline-2,3-dione (CNQX, 20 µM, AMPA antagonist) and dl-2-amino-5-phosphonopentanoic acid (APV, 100 µM, NMDA receptor antagonist).

Resting membrane potential (RMP) was measured as the membrane potential baseline value obtained in current-clamp mode in the absence of current injection. The action potential (AP) threshold was measured offline using Clampfit 10.7. The current–voltage relationship experiments (to evaluate action potential firing rate) consisted of a series of current injections (500 ms duration) between − 20 and + 280 pA, delivered in 20 pA increments.

### Confocal imaging

Confocal images were captured on an Olympus FV3000RS microscope using resonant, one-way scanning at 512 × 512 resolution. The following fluorophores were used: Alexa Fluor 405 (DAPI), Alexa Fluor 488, Alexa Fluor 568, Alexa Fluor 647. Laser intensities, gains, and offsets were set at thresholds that reflect minimal fluorescence in respective control stains of sections incubated with only blocking buffer and secondary antibodies for each experiment. Imaging parameters were tailored for each experimental application but were kept consistent between genotypes and all other respective experimental and control stains; sections used for quantification of MS-GABA YFP + processes were imaged at 20x magnification with 2x zoom, step size 1 µM. Sections used for MS-YFP + cell density were imaged at 20x magnification with 1 µM step size.

### In-vivo fiber photometry

For in vivo photometry recordings, mice were unilaterally injected with 250 nl of AAV5-DIO-GCaM

followed by pseudotyped rabies virus

P6f mixed with AAV5-DIO-mCherry (5:1) into the hilus of the DG (AP: −2.0 mm, ML: +1.4 mm, DV: −2.0 mm) and MS (AP: −0.7 mm, ML: 0 mm, DV: −3.8 mm). After injection, optical fibers (Newdoon Inc, O.D.: 1.25 mm, core: 200 mm, NA: 0.37) were implanted above the DG (AP: −2.0 mm, ML: +1.4 mm, DV: −1.8 mm). Mice were allowed 2 weeks of recovery before behavioral testing and fiber photometry.

The multi-fiber photometry system was used as previously described [45]. We used a 488 nm, 40 mW continuous wave (CW) laser (OBIS 488 LS-60, Coherent, Santa Clara, CA) for both GCaMP and mCherry excitation. The laser was launched into a fluorescence cube (DFM1, Thorlabs, Newton, NJ), which contained a dichroic mirror (ZT405/488/561/640 rpcv2, Chroma Technology Corp, Bellows Falls, VT) to reflect and launch the combined laser into the core of a 105/125 mm core/cladding multi-mode optical fiber patch cable. The distal end of the patch cable was connected to the implanted optical fiber. The emission fluorescent signal was collected from the fiber, then traveled back along the same patch cable into the fluorescence cube, passed through the dichroic mirror and an emission filter (ZET405/488/561/640mv2, Chroma Technology Corp, Bellows Falls, VT), and launched into the core of an AR-coated multi-mode patch cable (M200L02S-A, Thorlabs, Newton, NJ). The AR-coated patch cable was connected to a spectrometer (QE Pro-FL, Ocean Optics, Largo, FL) for each acquisition channel.

Spectral data was acquired by OceanView software (Ocean Optics, Inc) at 10 Hz and was synchronized to a 20 Hz video recording system during animal behavior. In-vivo recordings were carried out in a 30 lx red light environment in either the open-top home cage (21.6 × 17.8 × 12.7 cm) or the NPR arena (45 × 45 × 45 cm). Photometry data were exported to MATLAB R2014b for analysis. 594 nm mCherry signals were used for motion control. Photometry signals (ΔF/F) were derived by calculating (F–F0)/F0, where F0 is the median of the fluorescence signal. The threshold of ΔF/F was calculated as 3 SD. Home cage recordings lasted 10 min per mouse, and ΔF/F > threshold was calculated as calcium activity events in the homecage [46, 47].

### Novel place recognition

Mice were acclimated to handling and testing arena over 5 days. During training, mice were familiarized to two unique objects secured to a white plexiglass arena (45 × 45 × 45 cm). After 24 h, one of the two objects was moved to a new location. Orientation and time spent with objects were calculated by head orientation towards the object within 2 cm proximity. These measurements were aggregated into recognition scores by subtracting the time spent exploring the novel-placed object by the time spent exploring the familiar (unmoved) place object and dividing this difference by the total time spent exploring both objects in the test (retrieval) phase To compensate for possible exploration reluctance, familiarization and test periods began when animals left arena corners to actively explore. When analyzed concurrently with in-vivo fiber photometry recordings, optical fiber cables were attached to head-fixed implants 5 min prior to behavioral analysis to reduce stress.

### Quantification and statistical analysis

Data were reported and presented as the mean ± SEM. N means animal number unless stated. The sample size was modeled after similarly published research. Both behavioral analysis and tracing analyses were performed blinded to the conditions of the experiments. Unpaired d t-tests were used unless specified otherwise. For the interaction of multiple variables including genotype, two-way ANOVA was used. For probability distribution analysis, Kolmogorov-Smirnov tests were used. For correlation analyses, the Pearson correlation test was performed. Testing was always performed as two-tailed with α = 0.05. ‘n.s’ indicates no significant difference (p > 0.05). Statistical analyses were performed in Prism 8 (GraphPad).

### Data and code availability

All electrophysiology, behavioral assay, and microscopy data reported in this paper will be shared by the lead contact upon request. Any additional information required to reanalyze the data reported in this paper is available from the lead contact upon request. This manuscript does not report the original code.

## Results

### DG interneurons exhibit high anatomical connectivity with MS GABAergic neurons 

**Fig. 1:**
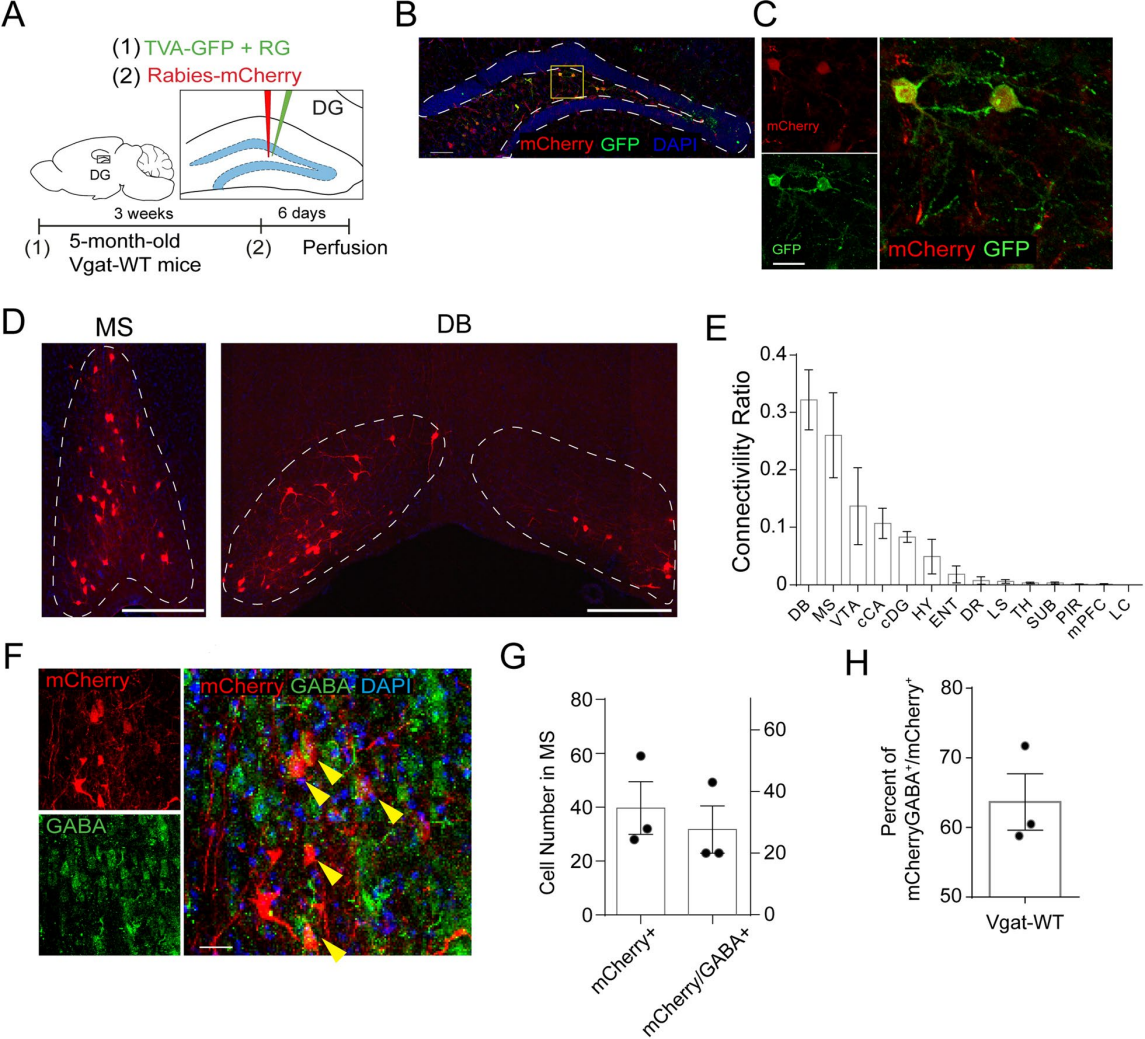
DG interneurons are highly connected with MS GABAergic neurons. **A** Schematic for monosynaptic retrograde tracing of GABAergic neurons in the DG of Vgat-Cre mice. **B** Representative images of mCherry-Rabies virus labeling in the DG of Vgat-Cre (scale bar = 200 μm). **C** Representative images of mCherry and GFP labeling in the starter cells from the retrograde tracing of DG GABAergic neurons (scale bar = 50 μm). **D** Representative images of mCherry labelied input cells in the MS and DB of 5 month Vgat-Cre mice. **E** Quantification of Vgat-Cre connectivity in 5 month Vgat-Cre mice. Data from 4 Vgat-WT mice. *DB *Diagonal band of Broca, *MS *Medial Septum, *VTA *Ventral Tegmental Area, *cCA *contralateral Cornu Ammonis, *cDG *contralateral Dentate Gyrus, *HY  *Hypothalamus, *ENT *Entorhinal cortex, *DR *Dorsal raphe, *LS *Lateral Septum, *TH *Thalamus, *SUB *Subiculum, *PIR *Piriform cortex, *mPFC *medial Prefrontal Cortex, *LC *Locus coeruleus. **F** Representative images of mCherry-labeled input cells in the MS co-labeled with anti-GABA antibodies (Yellow arrows indicate co-localization) in 5 month Vgat-Cre mice. (scale bar = 25 μm). **G**–**H** Quantification of mCherry^+^, mCherry^+^GABA^+^ cells and percentage of mCherry^+^GABA^+^ in total mCherry^+^ cells in retrograde labeling of MS neurons in 5 month Vgat-Cre mice. 3 mice and 3 slices/mouse were counted

To identify the inputs to DG GABAergic interneurons, we used a rabies-virus (RV) based approach for monosynaptic retrograde tracing [48–52] (Fig. 1A). First, we expressed the avian retrovirus-specific receptor TVA-EGFP and the rabies virus glycoprotein (RG) specifically in the DG interneurons of Vgat-Cre mice, followed by pseudotyped rabies virus expressing mCherry [40–42] (Fig. 1B). The DG interneurons (starter cells) were labeled by eGFP and mCherry (yellow cells, Fig. 1C). The whole-brain distribution of the long-range presynaptic inputs to dentate interneurons were aligned to identified brain regions based on Allen Institute’s reference atlas [53]. Our results revealed that RV-labeled mCherry + presynaptic input neurons are predominantly located in the MS and the diagonal band of Broca (DB) of the septal nuclei, as compared to other brain regions (Fig. 1D–E). The DB mainly harbors cholinergic neurons that project to the hippocampus, which has been widely studied in the context of AD. Here we focus on the understudied connections from MS to DG interneurons. Interestingly, we found that 64% of RV-labeled mCherry + neurons in the MS are GABA^+^ (Fig. 1F–H), suggesting that MS GABAergic neurons are the major presynaptic inputs to the DG interneurons.

### Activities of DG and MS GABAergic neurons are correlated during spatial memory encoding

**Fig. 2:**
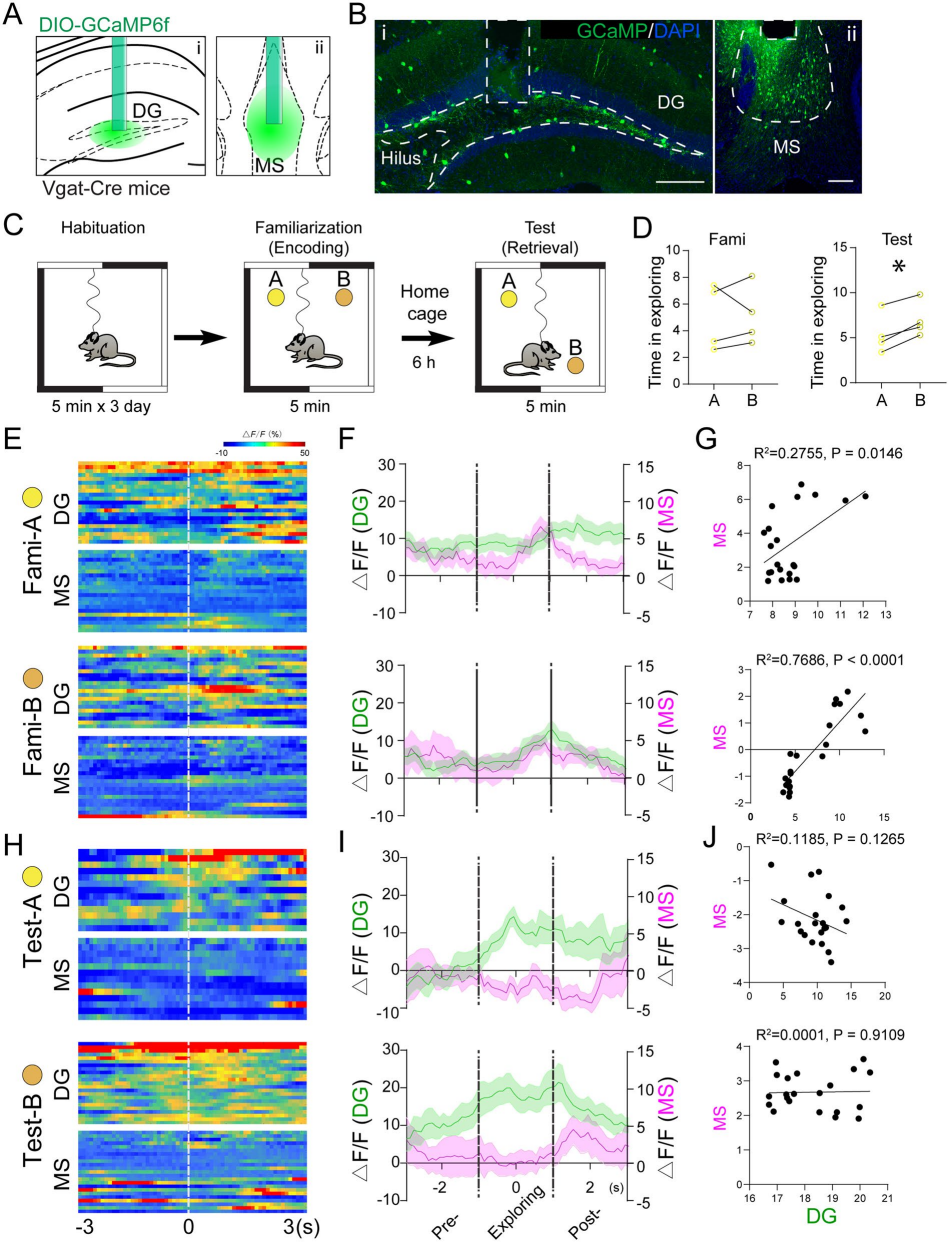
Activities of MS and DG GABAergic neurons correlate during spatial memory encoding. **A** VGAT-Cre animals with dual injection of AAV5-DIO-GCaMP6f and optical fiber implantation to the DG and MS. Enlarged implantation region schematics in the DG (i) and MS (ii). 4 Vgat-WT mice were included in analysis. **B** Representative images of GCaMP6f expression in the DG (i, scale bar = 200 μm) and MS (ii, scale bar = 100 μm). **C** Diagram of Vgat-Cre animals trained and tested in the NPR. **D** Time spent in exploring object-A and object-B during familiarization and test phase in the NPR test. n = 4 mice, paired t-test, *p < 0.05. **E** Scaled heat maps of DG and MS GABA neuron activity distribution when approaching objects A and B during the familiarization phase of the NPR test. **F** Averaged calcium activity of DG (green, left) and MS (magenta, right) GABA neuron activity pre, during, and post exploration of objects A and B during the familiarization phase of the NPR test. **G** Pearson correlation analysis of MS and DG GABA neuron calcium activity during interactions with objects A and B during the familiarization phase of the NPR test. Data points represent time-locked exploration events during NPR trials. **H** Scaled heat maps of DG and MS GABA neuron activity distribution when approaching objects A and B during the retrieval phase of the NPR test. **I** Averaged calcium activity of DG (green, left) and MS (magenta, right) GABA neuron activity pre, during, and post exploration of objects A and B during the test phase of the NPR trial. **J** Pearson correlation analysis of MS and DG interneuron calcium activity during interactions with objects A and B during the retrieval phase of the NPR test. Data points represent time-locked exploration events during NPR trials

Both DG interneurons and MS GABA neurons have been shown to be involved in spatial memory [54]. We therefore sought to determine the relationship between the activities of DG and MS GABAergic neurons during spatial memory tasks. Toward this direction, we performed in vivo fiber photometry recording in Vgat-Cre mice injected with AAV5-DIO-GCaMP6f to the MS and DG (Fig. 2A–B). We simultaneously recorded Ca^2+^ activities of VGAT + GCaMP + neurons in both the DG and MS during distinct phases of the novel place recognition (NPR) test: familiarization (memory encoding), when mice initially explore both objects A and B and form spatial memories of their locations; and test (memory retrieval), when mice retrieve spatial memory of both objects A and B after object B has been shifted to a novel place (Fig. 2C–D).

We analyzed the time-locked Ca^2+^ dynamics of DG and MS GABAergic neurons before, during, and after interactions with objects A and B during distinct phases of the NPR test. During the encoding phase, we found that Ca^2+^ dynamics in DG interneurons are highly correlated with that in MS GABAergic neurons (heat map of individual trials shown in Fig. 2E, averaged activity shown in Fig. 2F). Pearson analysis confirmed such correlation when mice were exploring both objects in the encoding phase of the NPR test (Fig. 2G). In contrast, during the retrieval phase, DG interneuron Ca^2+^ activity increased during the exploration of both objects A and B, but the activity of MS GABAergic neurons remained unchanged (averaged in Fig. 2I, heatmap of individual recordings in Fig. 2H, Additional file 1: Fig. S1). Pearson analysis showed no correlation in activities of MS and DG GABA neurons during the test phase of NPR (Fig. 2J). These results indicate that activities of DG and MS GABAergic neurons are correlated during the spatial memory encoding phase, but not the retrieval phase.

It has been shown that 3xTg-AD mice exhibit spatial memory deficits during the NPR test [55]. Therefore, we sought to address whether the correlation of DG and MS GABAergic activities was altered in AD mice during the NPR test. We crossed 3xTg-AD mice to Vgat-ires-Cre mice to generate Vgat-Cre::3xTg-AD^+/+^ mice (detailed in the "Methods" section, referred as Vgat-AD mice hereafter). Vgat-Cre mice were used as controls (Vgat-WT mice). We tested spatial memory performance in 6 months old Vgat-WT and Vgat-AD mice during NPR test, similar to the paradigm in Fig. 2C. Consistent with recent studies [55], 6 months old Vgat-AD mice exhibited decreased discrimination ratio when compared to Vgat-WT controls, indicating spatial memory deficits in early-stage AD (Additional file 1: Fig. S2A–C). Notably, Vgat-AD mice displayed higher reluctance to explore than their Vgat-WT counterparts, as measured by a significantly longer delay to initiate object exploration (Additional file 1: Fig. S2D), indicative of a mild anxiety phenotype previously observed in this model [56] and common in early-stage or prodromal dementia patients [57]. Next, we delivered the AAV-DIO-GCaMP6f to the DG and MS of the Vgat-WT/AD mice and recorded Ca^2+^ activities of DG and MS GABAergic neurons. Unfortunately, when connected to fiber photometry cables in the exploration arena, AD mice exhibited a significantly higher reluctance to explore the objects than WT controls during the NPR test at both memory encoding and retrieval phases. As a result, many mice failed to explore both objects during familiarization (encoding), thus disqualifying them from analysis, so we were not able to obtain sufficient trials to analyze Ca^2+^ activities in behaving AD mice.

### DG interneurons exhibit hyperactivity during early AD

**Fig. 3:**
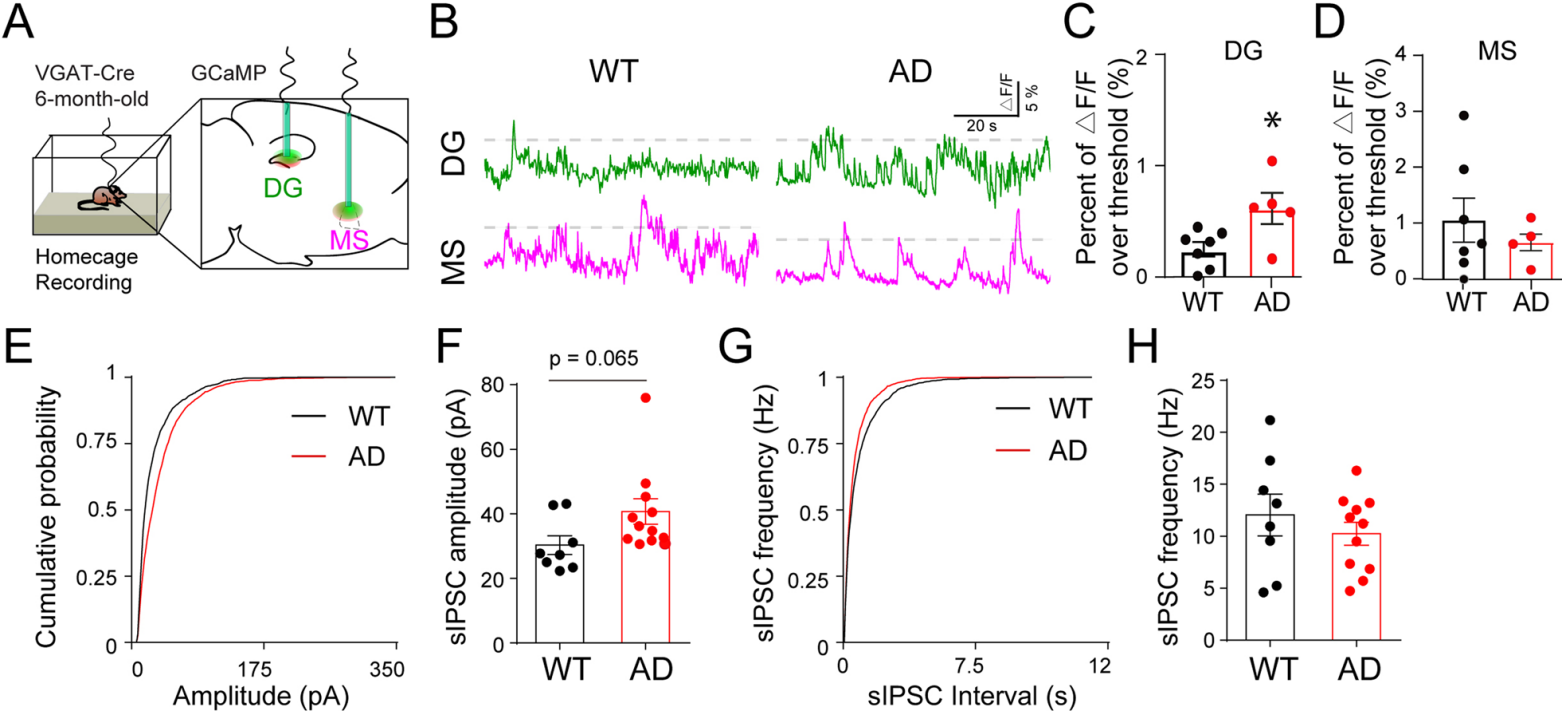
DG interneurons exhibit hyperactivity during early-stage AD. **A** Scheme of AAV injection and optical fiber implantation in the DG and MS of 6 month Vgat-WT and Vgat-AD mice. **B** Representative calcium activity traces of DG (green) and MS (magenta) GABAergic neurons in Vgat-WT and Vgat-AD mice during home cage recording. The dotted lines indicated the 3 SD thresholds. **C** Quantification of calcium activity in DG interneurons during home cage recordings in Vgat-WT and Vgat-AD mice. p < 0.05, n = 7, 5 mice. **D** Quantification of calcium activity in MS GABA during home cage recordings in Vgat-WT and Vgat-AD mice. n.s., n = 7, 5 mice. **E** Cumulative distribution of GC sIPSC amplitude in Vgat-WT and Vgat-AD mice. **F** Mean amplitude of GC sIPSCs in Vgat-WT and Vgat-AD. p = 0.065, n = 8, 12 cells. **G** Cumulative distribution of GC sIPSC interval in Vgat-WT and Vgat-AD mice. **H** Mean frequency of GC sIPSCs in Vgat-WT and Vgat-AD. p = 0.43, n = 8, 11 cells

Next, we sought to examine the vulnerability of the MS-DG GABAergic circuit during AD progression. Previous studies have identified pTau pathology in DG interneurons in 6 month 3xTg-AD mice [55]. In contrast, no intracellular or extracellular Aβ deposition was found in DG at this stage (Additional file 1: Fig. S2E), indicative of an early disease stage. We next addressed whether the MS-DG GABAergic circuit was altered during this disease stage. Given that Ca^2+^ activities in DG and MS GABAergic neurons could not be obtained during behavior in AD mice, we recorded Ca^2+^ activities of DG and MS GABA neurons in freely moving Vgat-WT and Vgat-AD mice in their home cages (Fig. 3A–B). Interestingly, we found that DG interneuron activity was significantly elevated in Vgat-AD mice when compared to Vgat-WT controls (Fig. 3C). In contrast, the activity of MS GABAergic neurons did not show significant alteration between Vgat-WT and Vgat-AD mice (Fig. 3D). These results indicate the selective vulnerability of DG interneurons during early AD.

To address whether DG interneuron hyperactivity induces loss of DG interneurons, we examined the density of DG GABAergic neurons by immunohistology using an anti-GABA antibody (Additional file 1: Fig. S2F). We found no change in the density of DG GABA^+^ neurons when comparing Vgat-AD and Vgat-WT mice (Additional file 1: Fig. S2G). These data suggest that DG interneuron hyperactivity is not associated with overt loss of DG interneurons during early-stage AD.

### DG interneuron hyperactivity coincides with synaptic remodeling of inhibitory inputs onto granule cells during early-stage AD

We next sought to address whether DG GABAergic interneuron hyperactivity correlates with altered GABA transmission of these DG interneurons in early-stage Vgat-AD mice. Given that the major inhibitory inputs onto the DG granule cells (GCs) are from local interneurons, we recorded the spontaneous inhibitory postsynaptic currents (sIPSCs) in GCs of early-stage Vgat-AD mice as a readout for GABAergic transmission. We hypothesized that DG interneuron hyperexcitability should correlate with increased inhibitory inputs onto DG GCs. As a result, we observed a trend of increased mean amplitude of sIPSCs in GCs (p = 0.065) (Fig. 3E–F). The mean frequency of sIPSCs was unchanged (Fig. 3G–H). These results suggest that the inhibitory inputs onto GCs are increased, likely as a result of DG interneuron hyperexcitability.

We also measured other electrophysiological characteristics of DG GCs and found no differences in membrane capacitance (Additional file 1: Fig. S3A), time constant (Additional file 1: Fig. S3B), or access resistance (Additional file 1: Fig. S3C). We did, however, detect significantly increased membrane resistance in Vgat-AD mice as compared to Vgat-WT controls (Additional file 1: Fig. S3D), suggesting fewer open ion channels in early-stage AD.

### Structural remodeling in MS-DG GABAergic circuits during early AD

**Fig. 4:**
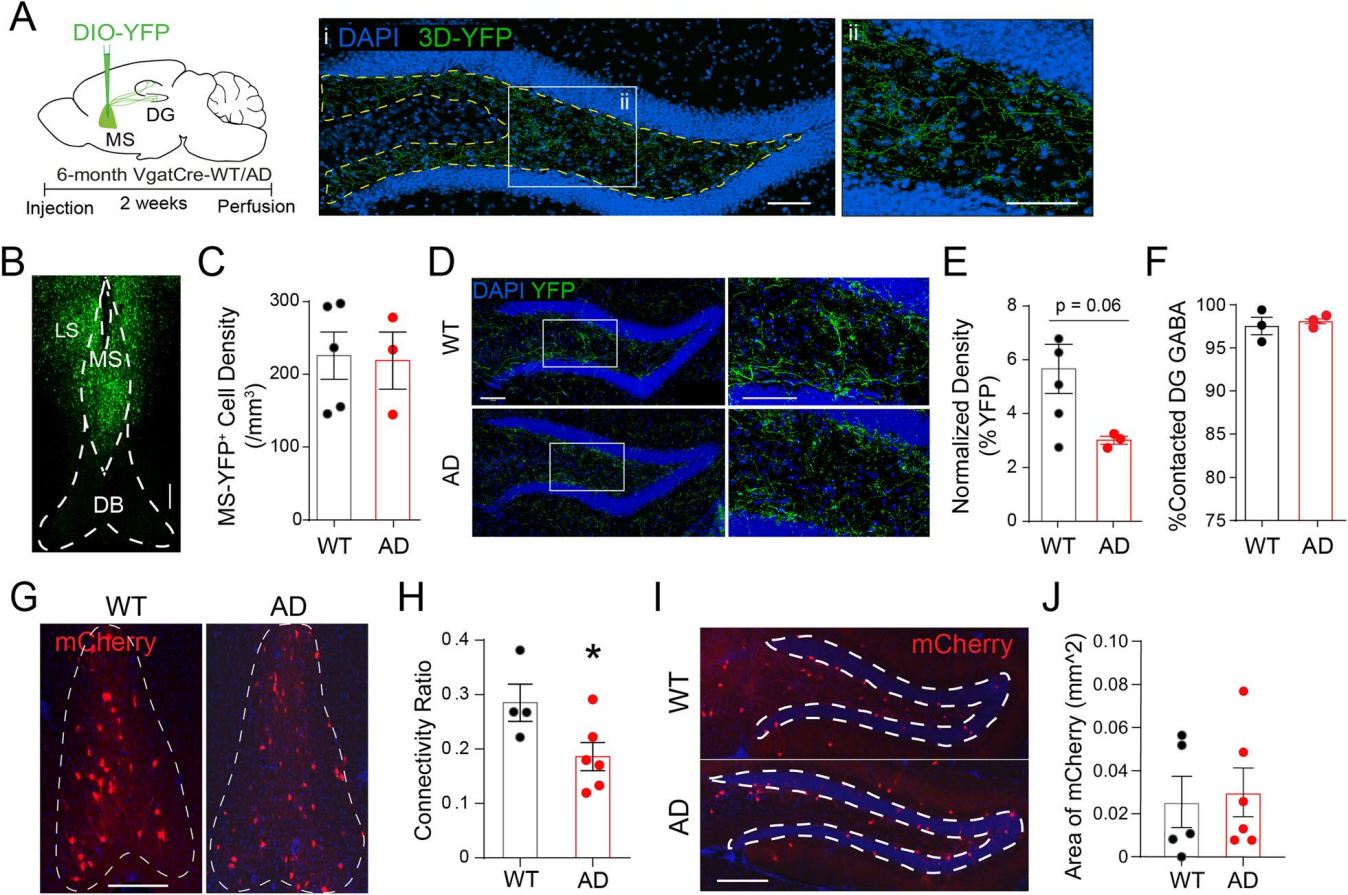
Structural remodeling of MS-DG GABAergic projections during early-stage AD. **A** Injection scheme and 3D reconstruction example for anterograde tracing of MS GABAergic projections to the hilus of 6 month Vgat-WT and Vgat-AD mice. Scale bar = 100 μm. **B** Representative images of YFP + neurons in the MS. Scale bar = 100 μm. **C** Quantification of YFP-labeled cell density in the medial septum of Vgat-WT and Vgat-AD mice at 6 months of age. p = 0.90, n = 5, 3 mice. **D** Representative images of MS-GABA projections to the DG of 6 month Vgat-WT and Vgat-AD mice. Scale bar = 100 μm. **E** Quantification of YFP + GABAergic septo-DG projections in the hilus of 6 month Vgat-WT and Vgat-AD mice. n = 5, 3 mice, p = 0.06. **F** Quantification of % DG GABA^+^ cells contacted by YFP^+^ GABAergic septo-DG projections in the hilus of 6 month Vgat-WT and Vgat-AD mice, n = 3, 3 mice, p = 0.6325. **G** Representative images of mCherry^+^ input cells in MS of 5 months old Vgat-WT and Vgat-AD mice. Scale bar = 300 μm. **H** Quantification of mCherry^+^ input cells in highlighted regions of interest in 5 month Vgat-WT and Vgat-AD mice. n = 4, 6 mice, p = 0.463. **I** Representative images of retrograde tracing injection sites (DG) in 5 month Vgat-WT and Vgat-AD mice. Scale bar = 300 μm. **J** mCherry^+^ area within the injection site ROI (hilus) in 5 month Vgat-WT and Vgat-AD mice. p = 0.647

A recent study measured GABAergic responses in vivo with iGABASnFR, a genetically engineered GABA sensor, in 6 months old 3xTg-AD mice, and found reduced GABAergic responses in the hilus, as compared to Vgat-WT mice [55], suggesting impaired GABA transmission during early AD. Since MS GABA neurons send abundant projections to the hilus and are the major GABAergic inputs to the local interneurons, we wondered whether MS GABAergic inputs onto DG interneurons are reduced, which could contribute to the observed hyperactivity in DG interneurons during early AD (Fig. 3B). To test this possibility, we performed anterograde tracing of MS-DG GABAergic projections by injecting Cre-dependent AAV2-DIO-YFP into the MS of 6 months old Vgat-WT and Vgat-AD mice (Fig. 4A). We performed 3D volumetric reconstruction using IMARIS to quantify the density of YFP^+^ projections in the hilus of Vgat-WT and Vgat-AD mice (Fig. 4A i, ii). We normalized the density of DG YFP^+^ processes in relation to YFP^+^ cell density in the MS (Fig. 4B–C) and found a trend toward lower densities of MS-DG projections in AD mice as compared to Vgat-WT controls (p = 0.06) (Fig. 4D–E). These results suggest that MS-DG projections begin to remodel in order to reduce the inhibition onto local interneurons, which potentially contributes to their hyperactivity. The percentage of DG-GABA^+^ cells was unchanged between Vgat-AD and Vgat-WT mice (Fig. 4F).

To further address whether there was altered anatomical connectivity between MS GABAergic neurons and DG interneurons in early-stage Vgat-AD mice, we performed RV-based monosynaptic retrograde tracing, similar to Fig. 1. We found that DG interneuron starter cell density in the DG was unchanged in 5 months old Vgat-WT and Vgat-AD mice (Fig. 4I–J). Then we calculated the connectivity ratio between MS mCherry^+^ input neurons and DG interneurons (starter cells) and found a significant reduction in connectivity in Vgat-AD mice when compared to Vgat-WT controls (Fig. 4G–J). These results suggest that the anatomical connectivity between MS and DG GABAergic neurons was significantly altered during early AD.

### Activation of MS-DG GABAergic projections increases the inhibitory inputs onto granule cells during early-stage AD

**Fig. 5:**
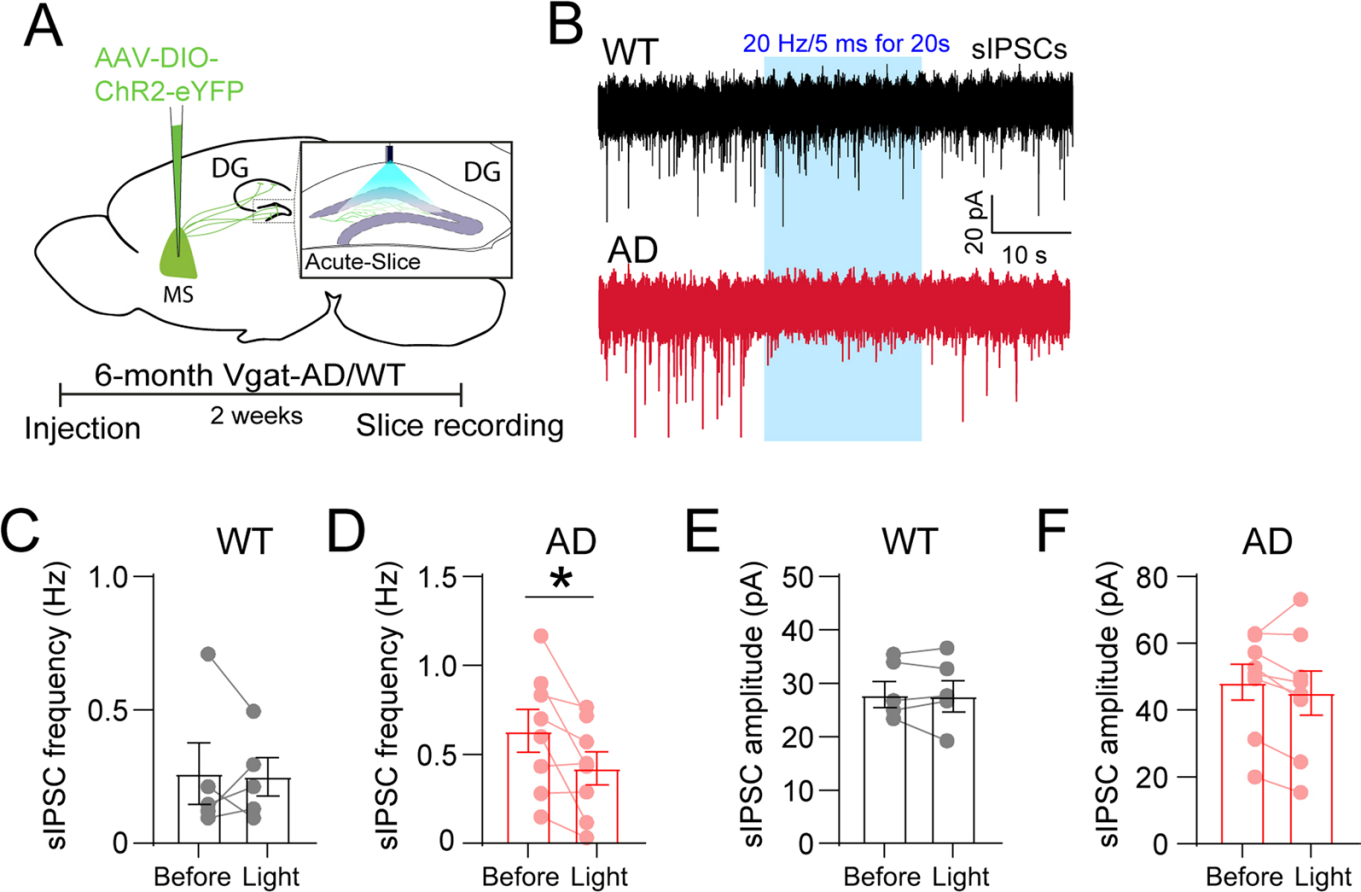
Activation of MS-DG GABAergic projections alters the inhibitory inputs onto GCs during early-stage AD. **A** Schematic of viral injection of ChR2 and blue light stimulation of septo-DG circuit projections to the hilus. **B** Representative traces of GC sIPSCs before, during (blue frame), and after blue light stimulation. **C** Quantification of sIPSC frequency in GCs before and during light stimulation in Vgat-WT mice, n = 5 cells. **D** Quantification of sIPSC frequency in GCs before and during light stimulation in Vgat-AD mice, n = 8 cells. **E** Quantification of sIPSC amplitude in GCs before and during light stimulation in Vgat-WT mice, n = 5 cells. **F** Quantification of sIPSC amplitude in GCs before and during light stimulation in Vgat-AD mice, n = 8 cells. *p < 0.05 by paired *t*-test. Error bars indicate SEM

To determine the functional properties of MS-DG projections in regulating GABAergic transmission in DG, we injected AAV5-DIO-YFP-ChR2 into the MS of 6 months old Vgat-WT or Vgat-AD mice (Fig. 5A) and recorded sIPSCs of GCs in response to optogenetic activation of MS-DG GABAergic projections in acute slice preparation (Fig. 5B). Interestingly, we found that optogenetic activation of MS-DG projections did not alter the mean frequency of sIPSCs in GCs of Vgat-WT mice (Fig. 5C) but resulted in a significant decrease of it in GCs of Vgat-AD mice (Fig. 5D). By contrast, optogenetic activation of MS-DG projections did not change the mean amplitude of sIPSCs in GCs of both Vgat-WT and Vgat-AD mice (Fig. 5E, F). These results suggest that increasing activity of MS-DG GABAergic circuits leads to larger inhibitory effects on DG interneurons in early Vgat-AD (vs. Vgat-WT) mice, thus reducing interneuron-mediated inhibitory inputs onto GCs. Such synaptic remodeling may serve as a compensatory mechanism to counteract the hyperactivity of DG interneurons during early AD by enhancing the strength of MS GABAergic inhibition onto DG interneurons.

### DG interneuron hyperactivity persists in late-stage AD and correlates with increased inhibition of dentate granule cells

**Fig. 6:**
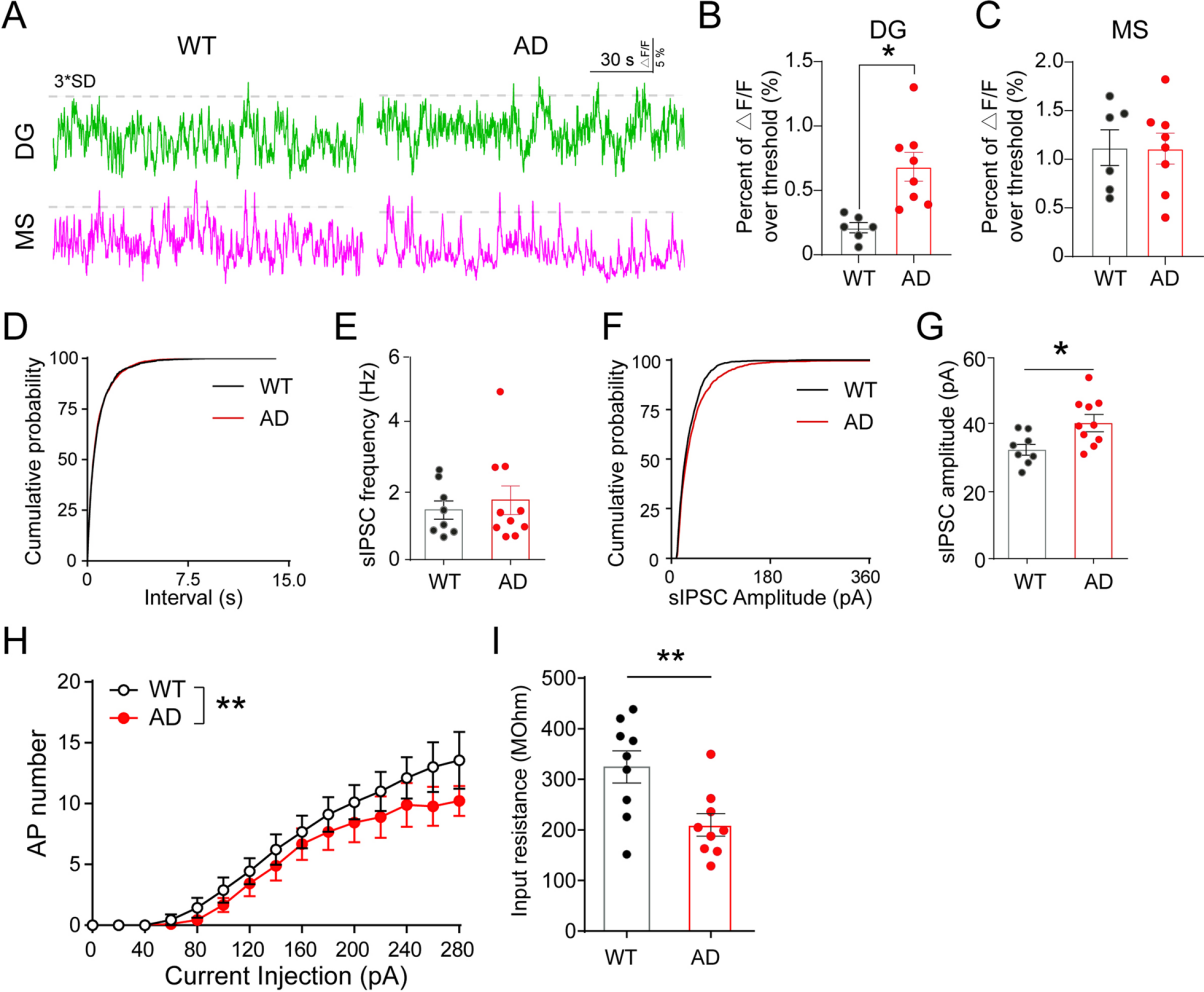
DG interneuron hyperactivity persists in late-stage AD and coincides with increased inhibition of dentate GCs. **A** Representative fiber photometry calcium activity traces of MS (magenta) and DG (green) GABA neurons in 14 months old Vgat-WT and Vgat-AD mice during home-cage recording. **B** Quantification of cumulative activity of DG interneurons in 14 months old Vgat-WT and Vgat-AD mice during home cage recordings. n = 6, 8 mice, p < 0.05. **C** Quantification of cumulative activity of MS GABA neurons in 14 month Vgat-WT and Vgat-AD mice during home cage recording. n = 6, 8 mice. **D** Cumulative distribution of sIPSC interval in GCs of Vgat-WT and Vgat-AD mice, p = 0.462. **E** Mean frequency of GC sIPSCs in Vgat-WT and Vgat-AD. p = 0.59, n = 8, 9 cells. **F** Cumulative distribution of sIPSC amplitude in GCs of Vgat-WT and Vgat-AD mice during acute slice electrophysiology, p < 0.0001. **G** Mean amplitude of GC sIPSCs in Vgat-WT and Vgat-AD mice. p = 0.01, n = 8, 9 cells. **H** Action potential distribution with various current steps in GCs of Vgat-WT and Vgat-AD mice. Two-way ANOVA, p = 0.003. **I** Quantification of GC input resistance in 14 month Vgat-WT and Vgat-AD mice. p = 0.009, n = 9, 9 cells

Having identified aberrant DG interneuron activity during early AD, we sought to examine the activity of DG interneurons at a later AD stage of 14 months of age (referred as late-stage AD) [37]. We selected this time point to model late-stage AD because 3xTg-AD mice started to develop various health issues after 14 months, which could potentially affect the synaptic and network aspects we examined [58]. We injected AAV-DIO-GCaMP6f into the DG and MS of 14 months old Vgat-WT and Vgat-AD mice and recorded Ca^2+^ activity in each brain region within the home cage as previously described (Fig. 3). Interestingly, we found that Ca^2+^ activity in DG interneurons (Fig. 6A–B), but not MS GABA neurons (Fig. 6C), was significantly elevated in Vgat-AD mice as compared to Vgat-WT controls. These results suggest that DG interneuron hyperactivity persists in late-stage AD.

Next, we examined whether DG GABAergic interneuron hyperactivity manifests in increased synaptic inhibitory events onto DG GCs in late-stage Vgat-AD mice. We recorded sIPSCs of GCs in both Vgat-WT and Vgat-AD mice and found no differences in the sIPSC frequency and interval (Fig. 6D–E) but did observe a significant increase in the amplitude of sIPSCs (Fig. 6F–G). Moreover, we found that the intrinsic excitability of GCs was significantly reduced in GCs of late-stage Vgat-AD mice (Fig. 6H). In addition, GCs in late AD mice exhibited a significant decrease in input resistance (Fig. 6I) and a trend towards reduced membrane potential (Additional file 1: Fig S4A), without altering the membrane capacitance (Additional file 1: Fig. S4B). These results support the persistence of DG interneuron hyperactivity and increased inhibitory inputs onto DG GCs as AD conditions progress to the late stages.

### DG interneurons exhibit higher anatomical connectivity with MS GABAergic neurons during late-stage AD

**Fig. 7:**
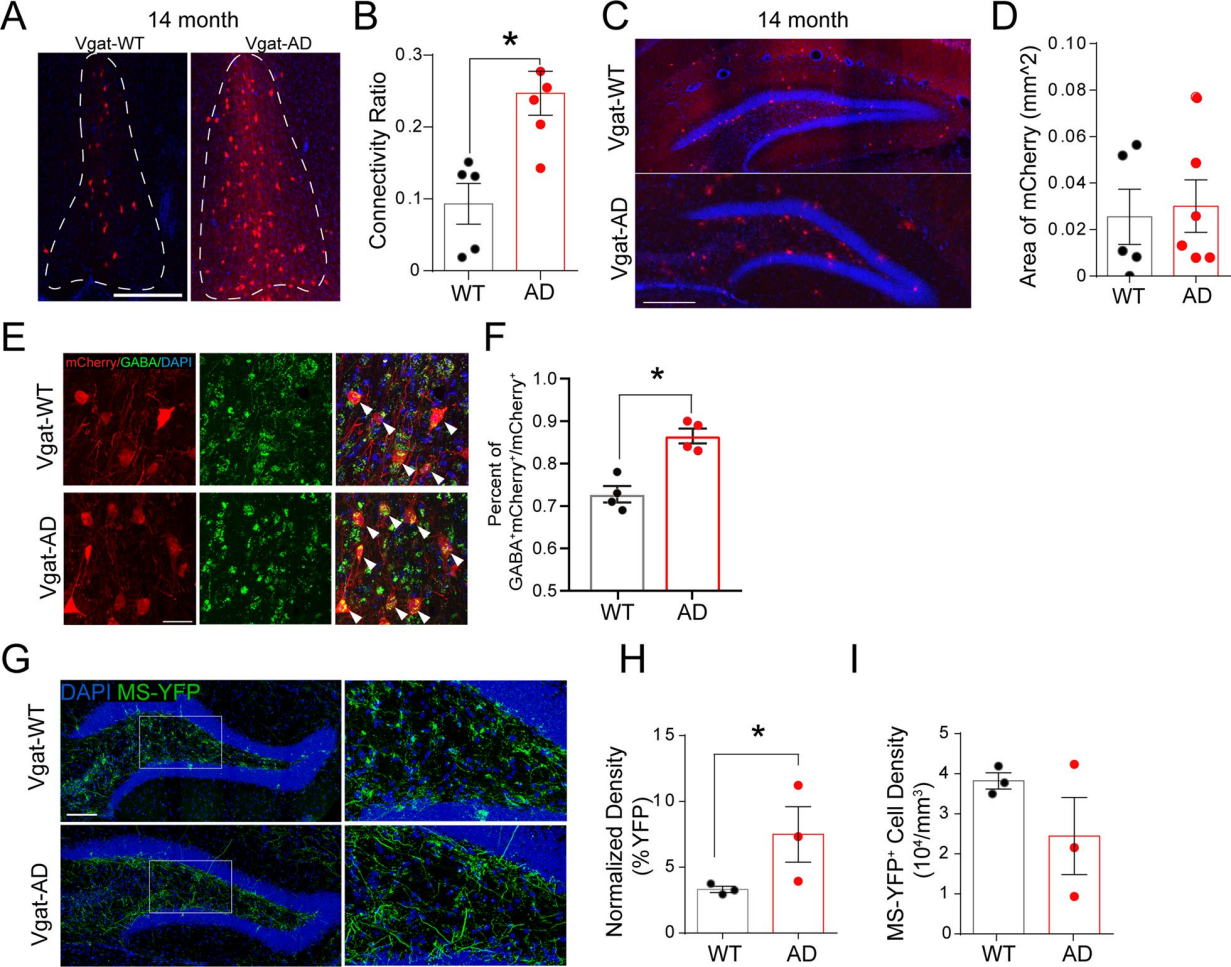
Compensatory structural remodeling of the MS-DG circuit in late-stage AD. **A** Representative immunofluorescence images showing mCherry input neurons in the MS of aged Vgat-WT and Vgat-AD mice. Scale bar = 300 μm. **B** Quantification of MS-DG connectivity ratio in 14 month Vgat-WT and Vgat-AD mice. n = 5, 5 mice. p = 0.002. **C** Representative images of retrograde tracing injection sites (DG) in 14 month Vgat-WT and Vgat-AD mice. Scale bar = 300 μm. **D** mCherry^+^ area within the injection site ROI (hilus) in 14 month Vgat-WT and Vgat-AD mice. n = 5, 6 mice. p = 0.96. **E** Representative dual immunofluorescence confocal images of the MS of aged Vgat-WT and Vgat-AD mice showing co-localization of mCherry input neurons co-localized with GABA staining. White arrows indicate co-localization. Scale bar = 30 μm. **F** Quantification of GABA^+^/mCherry^+^ neurons over total mCherry^+^ neurons in 14 month Vgat-WT and Vgat-AD mice. n = 4, 4 mice. p = 0.124. **G** Representative images of MS-GABA projections to the DG of 14 month Vgat-WT and Vgat-AD mice. Scale bar = 100 µm. **H** Quantification of MS GABA YFP^+^ process density in the hilus of 14 month Vgat-WT and Vgat-AD mice. n = 3, 3 mice, p = 0.0047. **I** Quantification of YFP-labeled cell density in the MS of Vgat-WT and Vgat-AD mice at 14 months of age. n = 3, 3 mice, p = 0.244

To address whether DG interneuron hyperactivity correlates with altered anatomical connectivity between MS and DG GABAergic neurons, we employed the previously described RV-based monosynaptic retrograde tracing in 14 months old Vgat-WT and Vgat-AD mice (similar to Fig. 1A). Interestingly, we found a significantly increased anatomical connectivity between MS GABAergic neurons and DG interneurons in Vgat-AD mice as compared to Vgat-WT mice (Fig. 7A–B). Specifically, double labeling of tissue sections from 14 months old WT and AD rabies virus-injected mice with anti-mCherry and anti-GABA antibodies (Fig. 7E) revealed a significant increase in the proportion of GABA^+^/mCherry^+^ cells in the MS of Vgat-AD mice when compared to Vgat-WT mice (Fig. 7F). Importantly, no alterations in the density of starter DG interneurons were observed (Fig. 7C–D). These results suggest higher anatomical connectivity between DG interneurons and MS GABAergic neurons, potentially as a compensatory mechanism to combat DG interneuron hyperactivity during late-stage AD.

### Excessive outgrowth of MS-DG GABAergic axons during late-stage AD

To further identify potential structural remodeling of MS-DG GABAergic projections during late-stage AD, we performed anterograde tracing in 14 months old Vgat-WT and Vgat-AD mice, as described previously (Fig. 5A). We analyzed the density of MS-DG YFP + projections in the hilus (Fig. 7G) using previously described 3D reconstruction techniques (Fig. 4A). Interestingly, we found a significant increase in the density of overall MS-DG YFP + projections (Fig. 7H) after normalizing for YFP^+^ cell density in the MS, which did not significantly differ between genotypes (Fig. 7I). These results indicate that excessive outgrowth of MS-DG projections occurs in late-stage Vgat-AD mice.

### Activation of MS-DG GABAergic projections has no effects on the inhibitory inputs onto GCs during late-stage AD

Given that MS-DG GABAergic projections are expanded in late-stage AD, we sought to analyze the downstream effects of this remodeling on DG GCs. To address whether these structural changes would lead to the alterations in synaptic strength of the MS-DG connections, we injected AAV5-DIO-YFP-ChR2 into the MS of 14 months old Vgat-WT or Vgat-AD mice and recorded sIPSCs in GCs upon optogenetic activation of MS-DG projections (similar to Fig. 5A). We found that opto-stimulation of MS-DG projections did not significantly alter the frequency or amplitude of sIPSCs in GCs in both Vgat-WT and Vgat-AD mice (Additional file 1: Fig. S4E–H, sample traces in Additional file 1: Fig. S4C–D). These results indicate that despite compensatory outgrowth of MS-DG GABA projections and increased anatomical connectivity of MS GABAergic neurons to DG interneurons, structural remodeling of the MS-DG GABAergic circuit does not exert functional effects on the inhibitory inputs to the GCs, presumably due to their inability to inhibit hyperactive DG interneurons in late-stage AD.

## Discussion

**Fig. 8:**
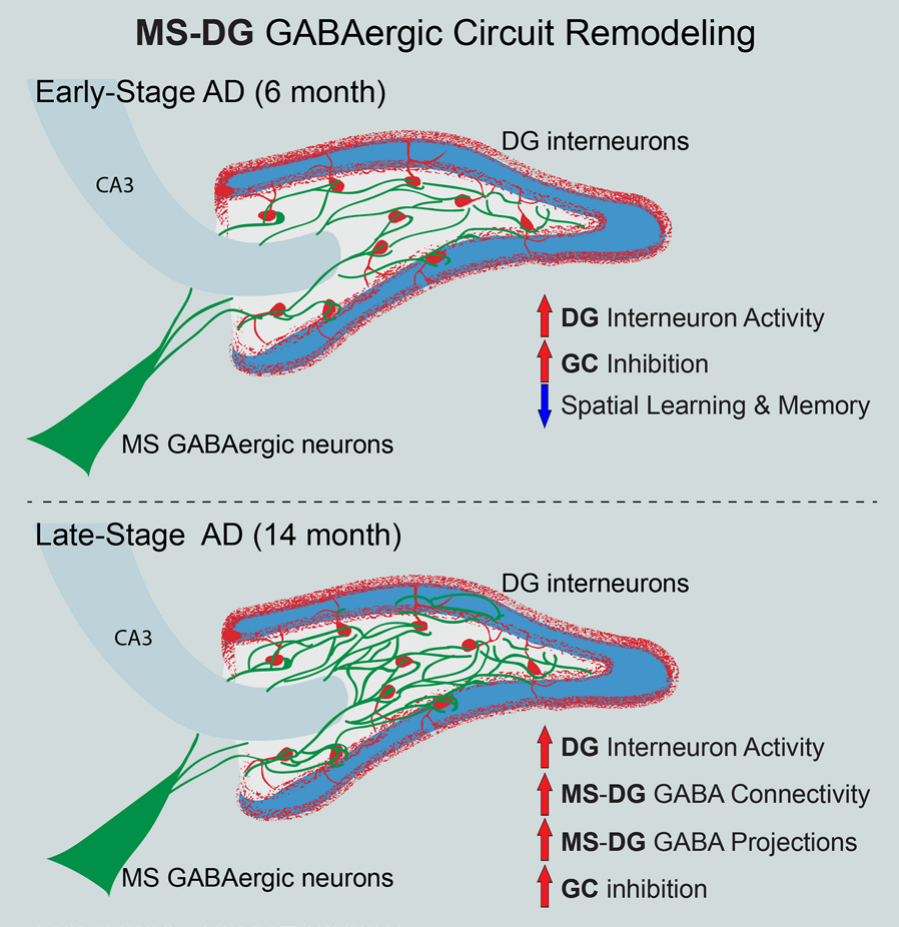
Dynamic remodeling of the MS-DG GABAergic network during AD progression. Distal MS GABAergic neurons develop a series of adaptive remodeling strategies to compensate for hyperactive DG interneurons during both early- and late-stage AD

In this study, we identified a long-distance GABAergic circuit that coupled the activities of highly connected MS and DG GABAergic neurons during spatial memory encoding. Interestingly, we found hyperactivity of DG interneurons in vivo starting from early AD and persisting throughout late AD stages. These findings are consistent with previous studies reporting CA1 interneuron hyperactivity in an amyloid-based AD mouse model [59, 60], thus supporting hippocampal interneuron dysfunction as a common AD hallmark. However, it remains unknown whether and how the distal neurons highly connected with hippocampal interneurons develop adaptive strategies to cope with these interneuron deficits. Such information is critically important to understand circuit and network vulnerability during AD progression. In this study, we reported the structural and functional remodeling of the MS-DG GABAergic circuit during disease progression in 3xTg-AD mice with dual amyloid/Tau pathology and slow disease progression. To address the vulnerability and subsequent remodeling of the MS-DG GABAergic circuit during AD progression, we employed a multi-faceted approach with a combination of circuit tracing, slice electrophysiology, and fiber photometry recording. Interestingly, we found that distal MS GABAergic neurons developed a series of adaptive strategies to compensate for DG interneuron hyperactivity. Specifically, upon activation, MS GABAergic neurons increased inhibitory synaptic strength onto DG interneurons during early-stage AD when AD pathology had not become apparent, followed by forming higher connectivity with DG interneurons along with aberrant outgrowth of MS-DG GABAergic projections during late-stage AD. However, these structural remodeling attempts failed to suppress the hyperactive DG interneurons in late-stage AD. It was recently reported that reduced GABA transmission in the DG of early-stage 3xTg-AD mice was associated with phospho-tau accumulation in DG interneurons [55]. Therefore, we speculate that such dynamic circuit-based remodeling reflects a compensatory mechanism to combat reduced GABA transmission by increasing interneuron activity during early AD, which subsequently leads to aberrant alterations of the MS-DG circuit to suppress hyperactive DG interneurons throughout AD progression (Fig. 8).

One interesting finding from our study is that DG interneuron hyperactivity exerts differential effects on the inhibitory inputs on GCs during early-stage versus late-stage AD. During early-stage AD, the DG interneurons in AD mice became hyperactive, potentially as a result of reduced DG GABA transmission which correlated with subtle alterations of the inhibitory inputs to GCs. Specifically, we observed decreased frequency distribution but increased amplitude distribution of sIPSCs in GCs of AD mice as compared to WT controls, without significant differences in mean frequency and amplitudes of sIPSCs. Interestingly, such DG interneuron hyperactivity persisted throughout AD progression, which correlated with significantly increased inhibitory inputs in GCs. In contrast to the hyperactivity of DG interneurons, we failed to observe activity change in MS GABAergic neurons during both early and late-stage AD. Moreover, significant structural remodeling of the MS-DG pathway was not observed until late-stage AD, despite the MS-DG GABAergic projections exhibiting a trend toward decrease during early AD. Specifically, higher anatomical connections between MS GABAergic neurons and DG interneurons and aberrant outgrowth of MS-DG GABAergic axons (sprouting) manifest only in late-stage AD.

Previous studies have shown that MS GABAergic neurons exclusively contact DG interneurons, which can be further delineated into diverse subtypes including parvalbumin (PV), somatostatin (SOM), neuropeptide Y (NPY), and cholecystokinin [61]. Furthermore, recent evidence showed specific isoforms of pTau in both dentate PV and SOM interneurons in early-stage [55]. Interestingly, our recent study showed that GABA exerts a depolarizing action on DG PV interneurons [26]. Therefore, it is possible that stimulating MS-DG GABAergic inputs simultaneously excites and inhibits DG PV^+^ and PV^−^ interneurons (such as SOM interneurons), respectively. This may explain why we failed to observe significant changes in the mean frequency and amplitude of sIPSCs in GCs of WT mice upon opto-stimulation of MS-DG GABAergic projections in our acute slice recording. However, this balance was disrupted in early AD mice, as we observed decreased frequency of sIPSCs in GCs. One explanation could be that MS-DG GABAergic inputs exhibit increased synaptic strength on DG PV^−^ interneurons during early AD, thus leading to a net effect of GC disinhibition. Such increased synaptic strength in MS-DG GABAergic pathway during early AD may serve as a compensatory mechanism to suppress hyperactive DG interneurons. Future studies that directly record distinct subtypes of DG interneurons in vivo are required to address selective vulnerability or functional loss of distinct DG interneuron subtypes during AD progression. Such information will guide circuit-based strategies to treat cognitive deficits in AD by targeting specific interneuron subtypes.

Our results highlight dynamic remodeling of the MS-DG GABAergic network during AD progression. Such adaptive remodeling may partially explain the slow progression and variable performance in cognitive tests observed in 3xTg-AD mice [62]. Whether this network remodeling occurs in other AD mouse models and human patients remains to be determined. In human patients, enlargement of MS was detected by magnetic resonance imaging (MRI) [63], indicating that monitoring MS structure could serve as a prognostic indicator for AD. Whether such alteration is mapped to the MS-DG GABAergic system remains to be determined.

## Conclusions

Our study reported the structural and functional remodeling of the MS-DG GABAergic circuit during disease progression in 3xTg-AD mice. Dynamic MS-DG GABAergic circuit remodeling represents a compensatory mechanism to combat DG interneuron hyperactivity induced by reduced GABA transmission.

## Supplementary Information

**Additional file 1: Figure S1.** Calciumactivity of MS and DG GABAergic neurons in NPR test. **Figure S2.** Spatial memory is impaired duringearly-stage AD without obvious AD pathological hallmarks in DG and MS. **Figure S3.** Electrophysiological characteristics ofgranule cells in early stage AD. **Figure S4.** Optogenetic stimulation of MS-DG circuitdid not change sIPSCs of GCs during late-stage AD. 

## Data Availability

All data generated or analyzed during this study are included in this published article and its additional information files. Any additional information required to reanalyze the data reported in this paper is available from the lead contact upon request.

## Abbreviations

AD: Alzheimer’s disease
MS: Medial septum
DG: Dentate gyrus
NPR: Novel place recognition
GC: Granule cells
sIPSCs: Spontaneous inhibitory postsynaptic currents
