# Supplementary material for: Compensatory remodeling of a septo-hippocampal GABAergic network in the triple transgenic Alzheimer’s mouse model
Source: J Transl Med. 2023 Apr 15;21:258. doi: 10.1186/s12967-023-04078-7 (PMC10105965; doi:10.1186/s12967-023-04078-7)
Supplement: Supplementary file 1 — Additional file 1: Figure S1. Calciumactivity of MS and DG GABAergic neurons in NPR test. Figure S2. Spatial memory is impaired duringearly-stage AD without obvious AD pathological hallmarks in DG and MS. Figure S3. Electrophysiological characteristics ofgranule cells in early stage AD. Figure S4. Optogenetic stimulation of MS-DG circuitdid not change sIPSCs of GCs during late-stage AD. [file 12967_2023_4078_MOESM1_ESM.docx]

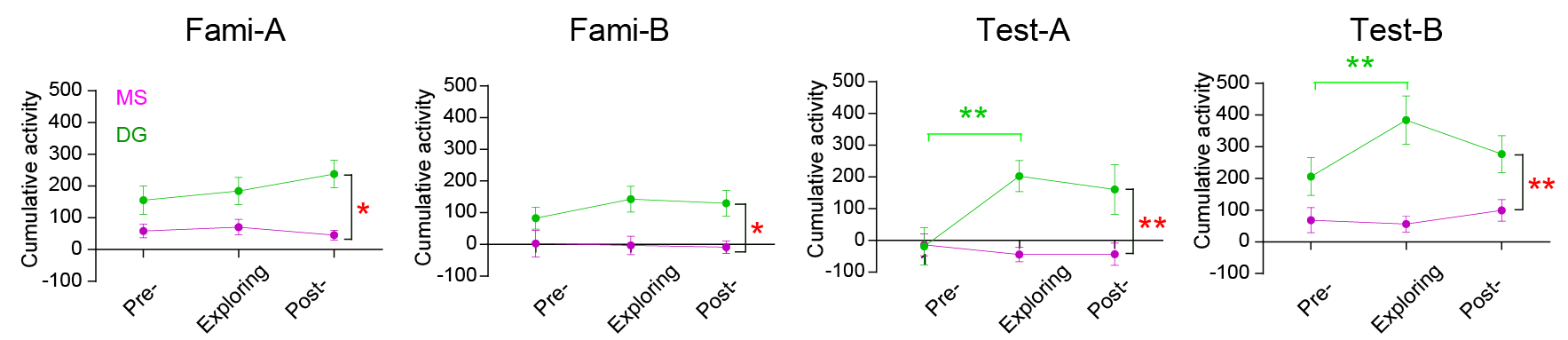


**Fig. S1** Calcium activity of MS and DG GABAergic neurons in NPR test.

Calcium activity of DG, but not MS, GABAergic neurons was increased in the memory recall in NPR test. n = 4 mice, paired t-test, * p < 0.05.


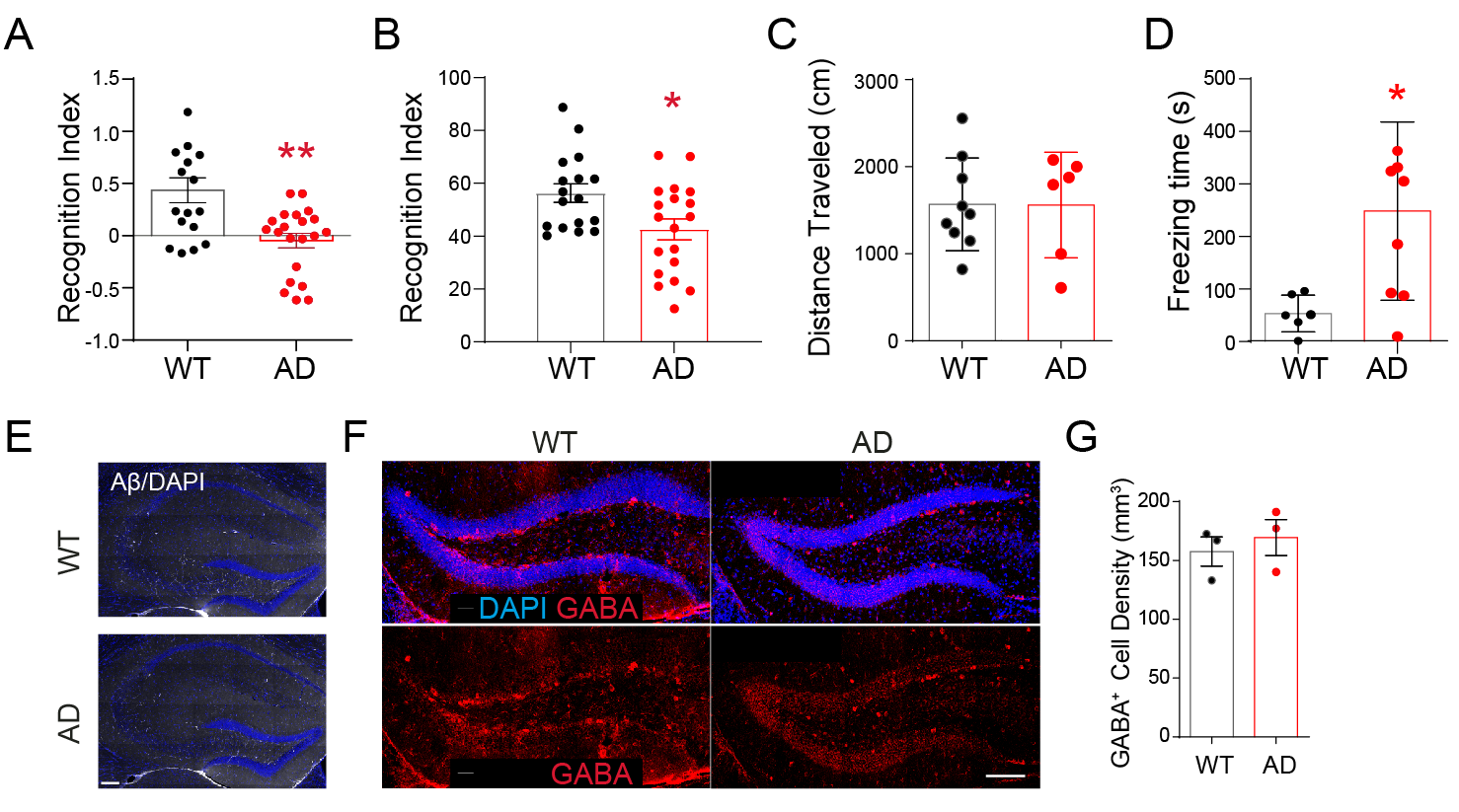


**Fig. S2 Spatial memory is impaired during early-stage AD without obvious AD pathological hallmarks in DG and MS.**

A) Quantification of 6 month Vgat-WT and Vgat-AD discriminative score (A-B/A+B) during the novel place recognition test, p = 0.0009, n = 17, 21 mice.

B) Recognition (B/A+B) of 6 month Vgat-WT and Vgat-AD in the NPR test. p = 0.0150, n = 17, 21 mice.

C) Traveled distance in the NPR test. n = 6-9 mice, P > 0.05.

D) Pre-NPR testing corner freezing time in a cohort of Vgat-WT and Vgat-AD mice, measured in seconds (s), p = 0.0319, n = 6, 8 mice

E) Representative confocal images of anti-amyloid (Aβ) in the hippocampus of 6 month WT and AD mice. Scale bar = 100 µm.

F) Representative confocal GABA immunofluorescence images in the DG of WT and AD mice at 6 months of age. Scale bar = 50 µm.

G) Quantification of DG GABA^+^ cell density. n = 3 mice, p = 0.278.

**
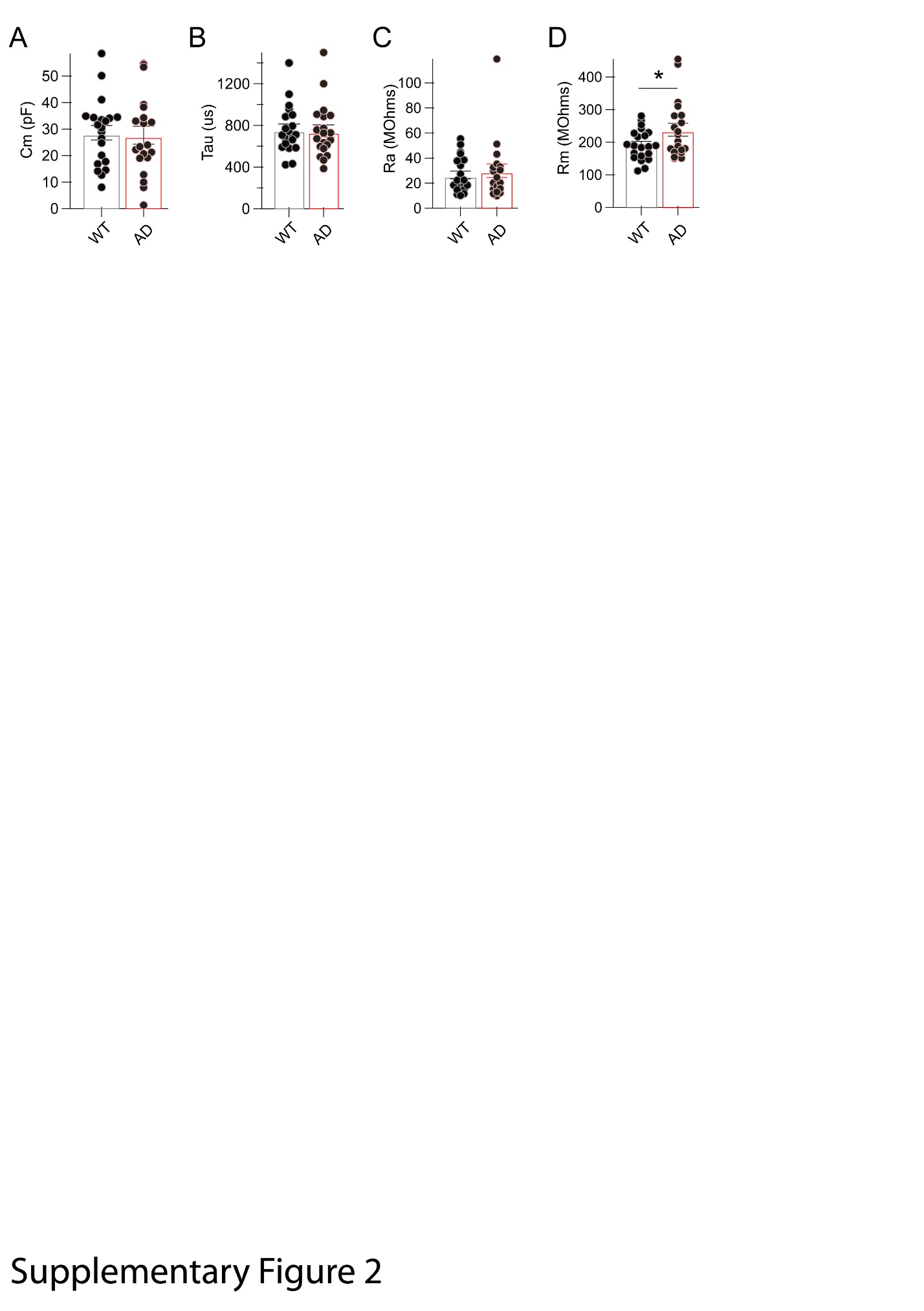
 Fig. S3 Electrophysiological characteristics of granule cells in early stage AD.**

A) Quantification of granule cell membrane capacitance in WT and AD mice, n = 21, 20 cells, p = 0.84.

B) Quantification of granule cell time constant in WT and AD mice, n = 21, 20 cells, p = 0.86.

C) Quantification of granule cell access resistance in WT and AD mice, n = 21, 20 cells, p = 0.58.

D) Quantification of granule cell membrane resistance in WT and AD mice, n = 21, 20 cells, p = 0.048.


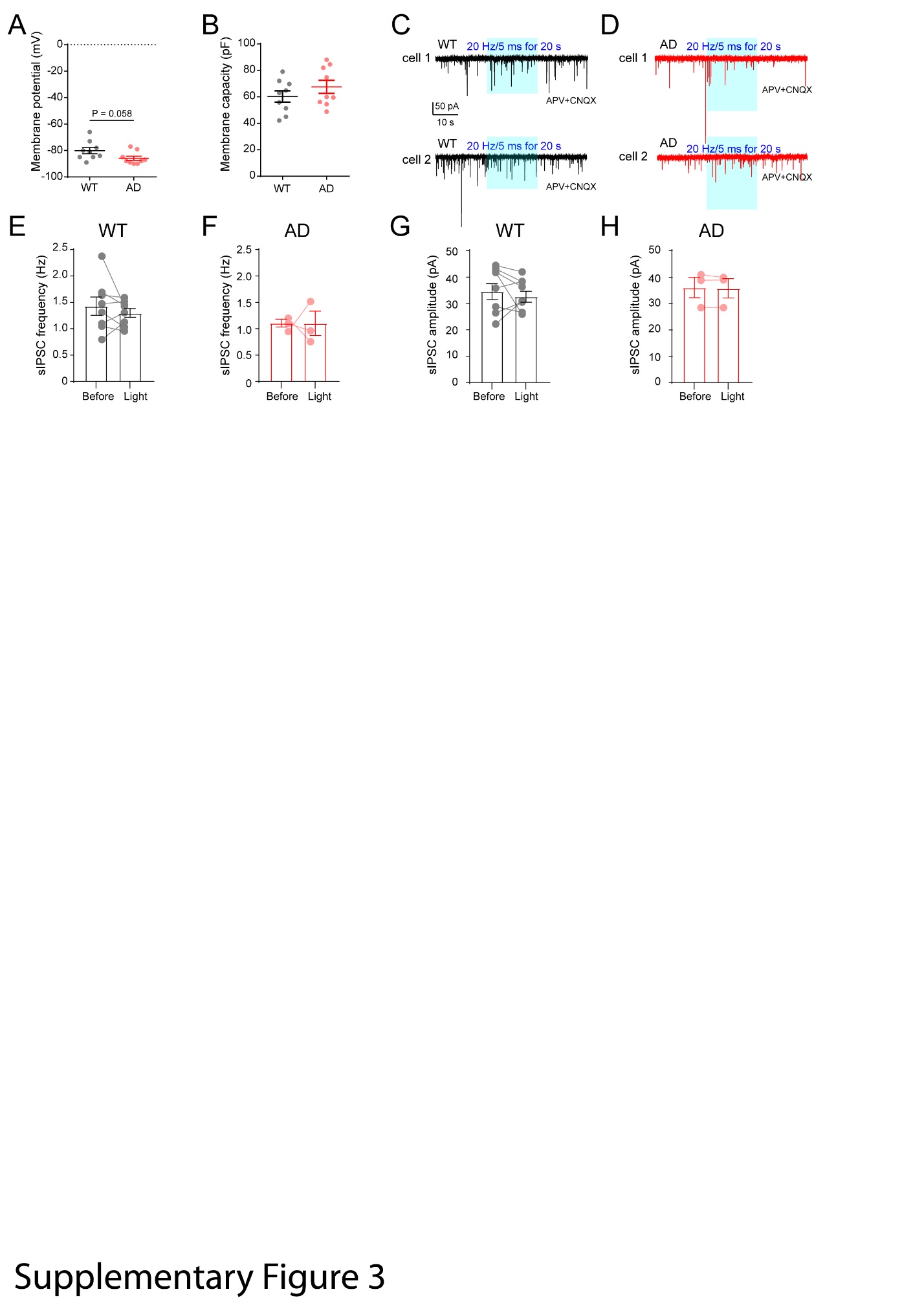


**Fig. S4 Optogenetic stimulation of MS-DG circuit did not change sIPSCs of GCs during late-stage AD.**

A) Quantification of GC membrane potential in 14 month WT and AD mice. n = 9, 9 cells, p = 0.058.

B) Quantification of GC membrane capacitance in 14 month WT and AD mice. n = 9, 9 cells, p = 0.278.

C) Representative 14 month WT GC traces during blue light stimulation (20 Hz/5 ms for 20 s) of MS GABA hilar projections in the presence of APV and CNQX.

D) Representative 14 month AD GC traces during blue light stimulation (20 Hz/5 ms for 20 s) of MS GABA hilar projections in the presence of APV and CNQX.

E) Quantification of GC sIPSC frequency before and during light stimulation in 14 month Vgat-WT mice, n = 8 cells.

F) Quantification of GC sIPSC frequency before and during light stimulation in 14 month Vgat-AD mice, n = 3 cells.

G) Quantification of GC sIPSC amplitude before and during light stimulation in 14 month Vgat-WT mice, n = 8 cells.

H) Quantification of GC sIPSC amplitude before and during light stimulation in 14 month Vgat-AD mice, n = 3 cells.
